# Identification of Conserved and Novel microRNAs from *Liriodendron chinense* Floral Tissues

**DOI:** 10.1371/journal.pone.0044696

**Published:** 2012-09-18

**Authors:** Kun Wang, Ming Li, Feng Gao, Shaoqing Li, Yingguo Zhu, Pingfang Yang

**Affiliations:** 1 Key Laboratory of Plant Germplasm Enhancement and Speciality Agriculture, Wuhan Botanical Garden of Chinese Academy of Sciences, Wuhan, China; 2 State Key Laboratory of Hybrid Rice, Wuhan University, Wuhan, China; 3 College of Life Science, Wuhan University, Wuhan, China; CNR, Italy

## Abstract

**Background:**

*Liriodendron chinense* (*L. chinense*) is an endangered basal angiosperm plant in China because of its low reproductive efficiency. Recently, miRNAs have obtained great attention because they can play important roles. Through high throughput sequencing technique, large amount of miRNAs were identified from different plant species. But there were few studies about the miRNAs in the basal angiosperms especially in the sexual reproduction process.

**Results:**

Deep sequencing technology was applied to discover miRNAs in *L. chinense* flowers at different stages. After bioinformatic analysis, 496 putative conserved miRNAs representing 97 families and 2 novel miRNAs were found. Among them, one is previously regarded as gymnosperm specific. Their expressions were further validated by Real-time PCR for 13 selected miRNAs. Putative targeting genes were predicted and categorized with gene ontology (GO) analysis. About ten percents of the targets are involved in the reproduction process. Further expressional analysis showed that many of these miRNAs were highly related to the reproductive growth.

**Conclusions:**

This is the first comprehensive identification of conserved and novel miRNAs in *L. chinense*. The data presented here might not only help to fill the gap of miRNA registered about basal angiosperm plants but also contribute to understanding the evolution of miRNAs. The differential expression of some of the miRNAs and the prediction of their target genes are also helpful in understanding the regulation of *L. chinense* sexual reproduction.

## Introduction

MicroRNAs are endogenous 21–24 nt small non-coding RNAs (sncRNAs) that have been found in a wide variety of organisms ranging from prokaryotes to eukaryotes [1], [2]. They negatively regulate gene expression on transcriptional and post-transcriptional level [3], and play pivotal roles in many aspects of plant growth and development [4], [5], [6] including floral organ identity, female gamete formation and reproductive development [7], [8]. In plant, most of the miRNA encoding genes are intergenic and rarely clustered in tandem. To broaden the knowledge of miRNAs, high throughput sequencing techniques have been applied and large amount of miRNAs from different plants were identified. Currently, 19 724 mature miRNAs belonging to 153 species are deposited in miRBase (release 17.0 version, April 2011) [9]. Among them, a total of 3362 are from 46 plant species. The majorities of these identified miRNAs are from monocots *Oryza sativa* and eudicots *Populus trichocarpa* and *Arabidopsis thaliana*. Other plants include algae *Chlamydomonas reinhardtii* [10] and *Porphyra yezoensis* [11], moss *Physcomitrella patens* [12], conifer *Pinus taeda* [13] and *Picea abies* [14], monocot *Brachypodium distachyon* [15], *Triticum aestivum* [16] and *Zea mays* [17], basal eudicot *Eschscholzia californica* [18], core eudicot *Aquilegia coerulea* [19], *Arachis hypogaea* [20], *Populus euphratica* [21], *Medicago truncatula* [22], *Glycine max* [23] and Euphorbiaceous plants [24]. However there is little information on the miRNAs from basal angiosperm plants except a study on the miRNAs from *Selaginella moellendorffii* [25].

**Figure 1:**
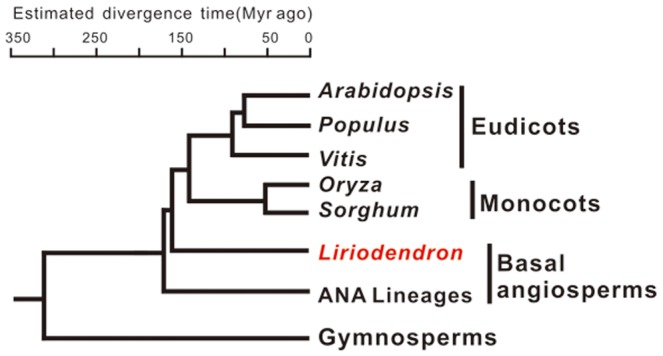
Phylogenetic position of Liriodendron genus (refer to literature [58]). ANA lineages stands for the genera Amborella, Nymphaea, Illicium, Trimenia and Austrobaileya.

*L.chinense* is one of the two species in Liriodendron genus which belongs to basal angiosperm. It is the early branching angiosperm lineages (Fig. 1). Because it occupied pivotal positions in the phylogenetic tree, the plant of Liriodendron genus was always used to study the evolution of flowering plants. Although basal angiosperms only represent 3% of whole angiosperm species, most of the diversity in floral structure and organization was found among them [26]. In addition, *L. chinense* is ideal for landscaping because of its beautiful flowers and leaves. But it is endangering in China, which is mainly resulted by its low sexual reproductive efficiency. The molecular mechanism underlying its sexual reproduction barrier is totally unknown. Many internal factors including miRNAs have been found to play regulatory roles in the reproductive growth of plants. To profile the miRNAs in the flowers of *L. chinense* may help to explore the mechanisms that control the sexual reproductive process in this species, and hence to overcome its barrier in reproduction.

To date, there is not any study about the miRNAs in *L. chinense* and no miRNAs in the miRBase were identified from this species. Based on previous studies, it is well known that plants use both conserved and species-specific miRNAs to function in different biological processes [25], [27], [28], [29]. A lot of miRNAs in Arabidopsis, nearly exactly match with those found in the gymnosperm Pinus genus [14], [30]. However, there are still a large number of species or genus specific miRNAs in an individual plant [29]. So to profile the miRNAs in more basal plant species will help to understand not only the evolution of miRNAs but also the regulation of different biological processes at different evolutionary stages. In this study, a deep sequencing of miRNAs was conducted in the flower of *L. chinense*. Because of the absence of its genome information, only 496 conserved and 2 novel miRNAs were identified, most of the reads were assigned as unknown small RNAs. These data can not only help to comprehensively understand the miRNAs expression profile of *L. chinense* and their roles in reproductive growth regulation, but also expand the knowledge of plant miRNAs as a whole.

## Results and Discussion

### Complex Small RNA Population in *L. chinense*

**Figure 2:**
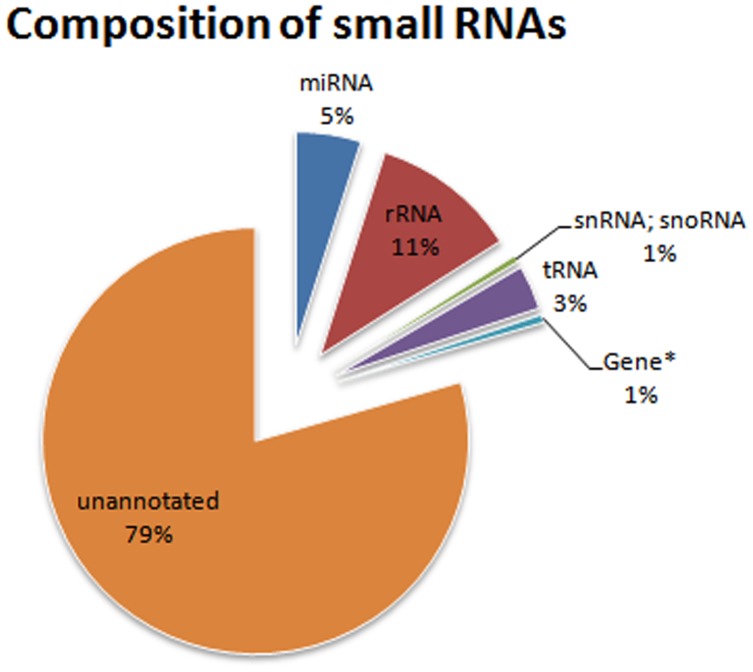
Composition of clean reads. The reads were annotated by Rfam.

**Figure 3:**
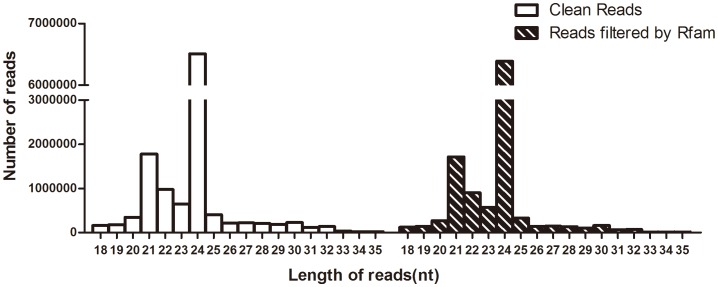
The length distribution of small RNAs. The open histogram at the left side shows the results from the total clean reads; the histogram with slash at the right side shows that the results after removing the reads for tRNA, rRNA, snRNA and snoRNA.

To profile the miRNAs in *L. chinense* at reproductive growth stage, a small RNA (18–30 nt) library from the mixed flower tissues was first constructed. After sequencing with Solexa sequencing instrument, a total of 15,353,485 raw reads were acquired (Table 1). Among them, 1,111,113 (7.27%) low quality, adaptors and contamination sequences were discarded. The rest 14,165,979 (92.73%) of clean reads represented 5,185,931 unique sequences. Except for the RNAs with known function (recognized by Rfam), a large amount of small RNAs with unknown function (unannotated by Rfam) were found in *L. chinense* (Fig. 2). With the availability of more and more genomic information, their function might be discovered in the near future.The size of these small RNAs ranged from 18 to 35 nt (Fig. 3 left side). The 24 nt small RNAs were the most abundant. They accounted for more than 50% of the total clean reads; the second abundant population was the 21 nt small RNAs, and the 22 nt ones were at the third place.

**Table 1:** Statistics of small RNA sequencing.

Category	Sequences generated
Raw reads	15353485
High quality reads	15277092(100%)
Sequences <18 nt reads	1057858
Adaptors only reads	47580
N value contained reads	5675
Clean reads	14165979(92.73%)
Unique sequence	5185931

Since the small RNAs might also include tRNA, rRNA, snRNA and snoRNA, further filtering by Blast with Rfam database was conducted to remove these small RNAs. After filtering, 696,864 miRNAs (5%) and 11,256,287 unannotated small RNAs (79%) were obtained. The ratio of tRNA, rRNA, snRNA and snoRNA was about 15% (Fig. 2). Actually, distribution pattern of the small RNAs’ size did not change after removing the tRNAs, rRNAs, snRNAs and snoRNAs (Fig. 3 right side), which implied that the small RNA library was with high quality.

The distribution pattern of the small RNA size in *L. chinense* was similar to that in other angiosperm species, such as Arabidopsis [31] and rice [30], which implied that *L. chinense* might possess similar processing components of small RNA biogenesis with rice and Arabidopsis. Whereas, the population of 24 nt RNAs was significantly low in conifer, which might be caused by the lack of DCL3 that mainly helps to produce 24 nt miRNAs in plants. It is reasonable in evolutionary history for their difference in the distribution pattern of small RNA. Because conifer is a gymnosperm plant, the other three are angiosperm plants. The comparison between basal angiosperm and gymnosperm species might help to understand the evolution of miRNAs biogenesis.

### Indentification of Conserved miRNAs in *L. chinense*

In *L. chinense,* there is no miRNA reported before. To identify the conserved miRNAs from our filtered data (the unique sequences except for the rRNA, snoRNAs, snRNAs and tRNAs), the sequences were aligned with all the miRNAs of Viridiplantae registered in miRBase 17. One or two mismatches were allowed during sequence alignment. Among the total 5,021,018 unique sequences input, 496 small RNAs matched to 97 known miRNAs families (Table S2) registered in miRBase. Among these 496 putative miRNAs, 102 had identical sequences and the rest had similar sequences with those miRNAs in the database (Table S2). These small RNAs were named as conserved miRNAs in *L. chinense*.

**Figure 4:**
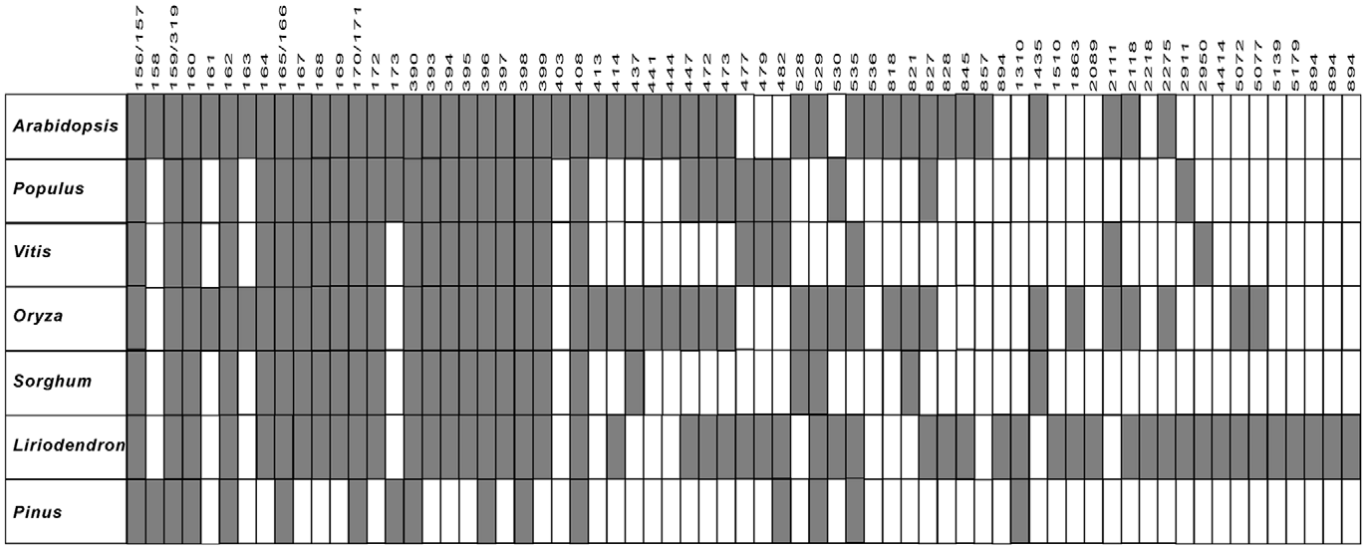
Comparison of some conserved miRNAs among *L. chinense* and other 6 plants. The rectangles filled with gray and white stand for “presence” and “absence” respectively. The 7 genera names were marked on the left and the miRNA families names was marked on the top and omit the prefix “miR”.

As we mentioned, this was the first comprehensive survey on miRNAs in basal angiosperm plants. To get a better understanding of the evolution of different miRNAs, the miRNA population in *L. chinense* was compared with those in other plants. Some representative genera including Pinus, Sorghum, Oryza, Vitis, Populus and Arabidopsis that stand for gymnosperms, monocots and eudicots plants were selected to conduct the comparison. Based on the statistic of miRBase and PMRD (http://bioinformatics.cau.edu.cn/PMRD/) and our results about *L. chinense*, we summarized the miRNA populations of 7 genera in figure 4. Twenty of the miRNAs exist in all the 6 angiosperm plant species; while only 10 of them also exist in pinus. As an important basal angiosperm species, *L. chinense* contains many valuable information to understand the evolution history of the miRNAs from gymnosperms to monocots and eudicots. For example, miR1310 is reported as a specific miRNA in gymnosperms plants [30], but it also existed in Liriodendron based on our results. This implied that miR1310 is not a gymnosperm specific miRNA. It might disappear during the evolution of the angiosperms. According to the current data, some miRNAs might only exist in eudicots and Liriodendron like miR477, miR479, miR482, miR828 and miR846 and some miRNAs might only exist in monocots and Liriodendron like miR1863. It still needs more data to explain when these moncot or eudicot specific miRNAs diverged during the evolution.

### Expression and Function Analysis of the Conserved miRNAs

**Figure 5:**
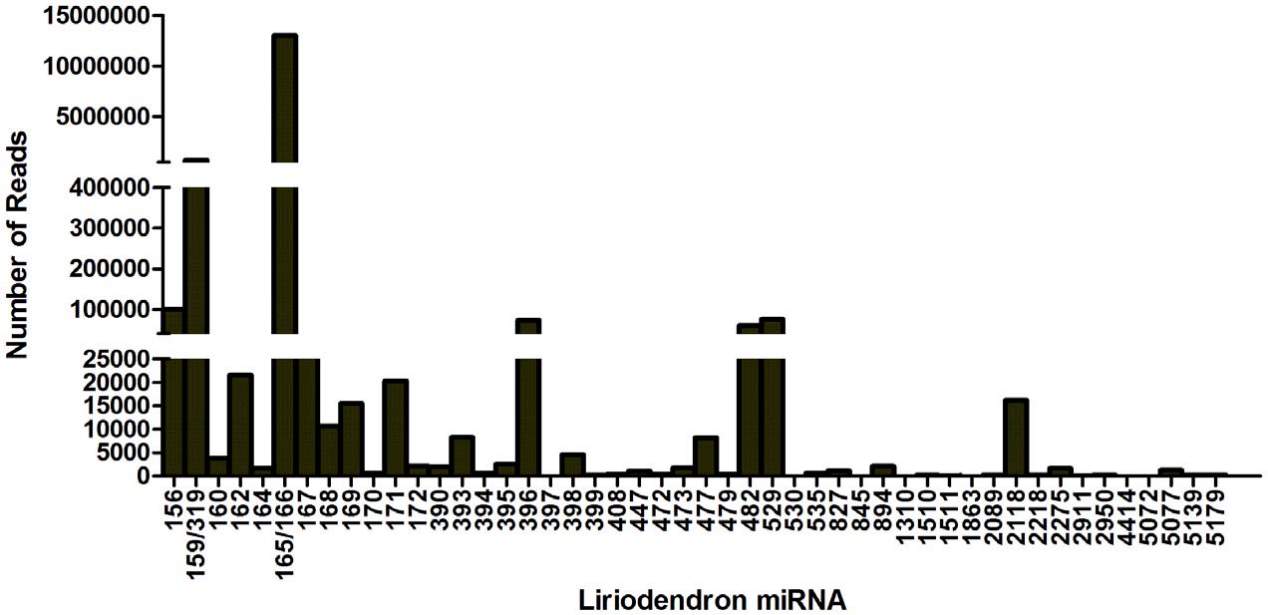
The abundance of some conserved miRNAs in flower of *L. chinense* based on sequencing data.

**Figure 6:**
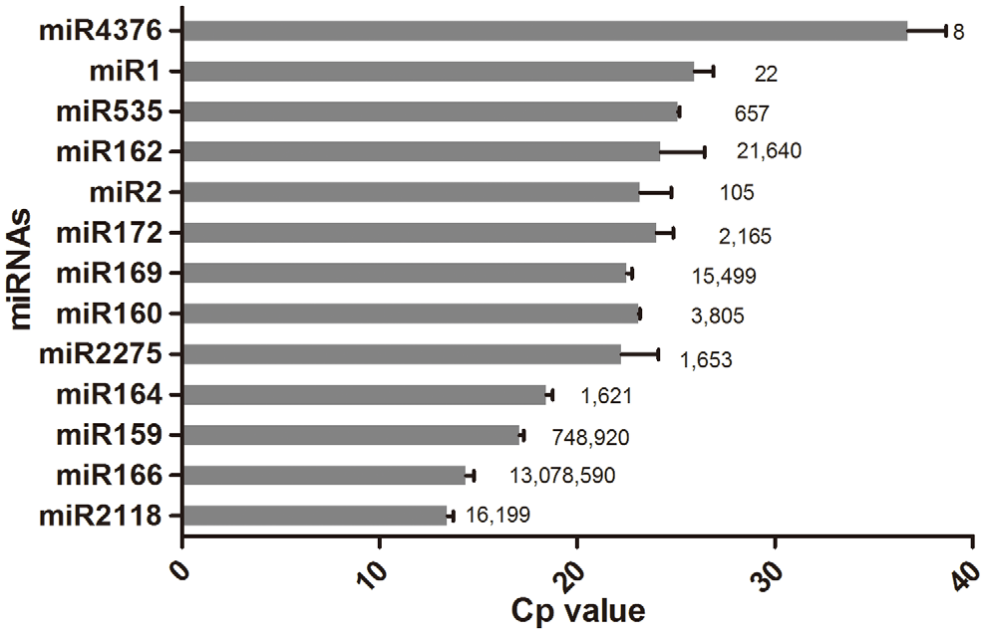
Real-time PCR validation of some miRNAs in *L. chinense*. the Cp value stands for the threshold cycle. Error bars indicate the standard deviation of three replicates. The corresponding sequencing reads number of individual miRNAs was on the right of columns and marked by rectangle.

The expression of plant miRNAs always appears to be spatio-temporal specific. To know their abundance in the flower of Liriodendron, the reads of 48 families of miRNAs that had more than 10 sequence counts were shown here (Fig. 5). The results showed that miR165/166, miR159/319, miR156, miR396, miR482, miR529, miR162, miR171, miR2118, miR169, miR168, miR393, miR 477, miR398, miR160, miR395, miR172, miR894, miR390, miR473, miR2275, miR164, miR5077, miR827 and miR447 had high abundance (reads counts >1000) in the flower of *L. chinense*. Amoung them, the more conserved miRNAs like miR165/166, miR159/319, miR156, miR396 and so on, tended to have higher abundance. However, the lower conserved miRNAs like miR477, miR447 and miR473 had relatively low abundance. To validate the sequencing data, some miRNAs were selected to do the real-time PCR. The Cp value from real-time PCR could reflect the abundance of miRNA at some extent when using equal amounts of RNA to perform stem-loop RT-PCR, the high Cp values obtained from the miRNA reflect its low level of expression and the low Cp values obtained from the miRNA reflect its high level of expression [20], [32], [33]. The RT-PCR results were largely consistent with the data from deep sequencing (Fig. 6). The miRNAs with high abundance (miR2118, miR166, miR159 and miR164) have low cp value (13.42±0.31, 14.36±0.41, 17.07±0.21, 18.43±0.32). As for the conflict between Real-time PCR and deep sequencing for miR2118, miR169 and miR162, it might be the results of the different amplification efficiency produced by different primers or other unknown reasons.

In order to explore the possible roles during the reproductive growth, we predicted the target genes of all the identified miRNAs by psRNATarget software (http://plantgrn.noble.org/psRNATarget) with ESTs of Liriodendron genus. The parameters were set as default during target genes searching. Many of the highly expressed miRNAs might have the conserved target genes, most of which were still transcription factors, similar with those in *Arabidopsis thaliana* or *Oryza sativa* (Table 2). On the contrary, targets of the lowly expressed miRNAs are not conserved during the evolution, such as miR160, miR164 and miR172. Functional categorization of the 1270 putative targets of the total predicted miRNAs in our study was also conducted to see if there was any propensity of the miRNA targets (Table S3). The proportion of transcription factor was low, which is different with that in maize [34]. This difference might be due to the evolution selection for the miRNA targets. Under natural selection pressure, the regulation between transcription factors and miRNAs were strengthened and accumulated in newly evolved species.

**Table 2:** Predicted targets of miRNAs that have high abundance in *L. chinense.*

MiRNA	Readscounts	Target in *At* *and os* 1	Putative target in *Liriodendron* 2
**447**	1114	2-PGK	Nucleotide binding protein
**827**	1165	ND	Phosphoribosyltransferase
**5077**	1302	ND	ND
**164**	1621	NAC	ND
**2275**	1653	ND	Prolyl oligopeptidase protein
			PPR protein
**473**	1754		ND
**390**	2091	ta-siRNA	Binding protein
**894**	2118		ND
**172**	2165	AP2	Kinesin
			Ribosomal protein
**395**	2513	APS	Vacuolar sorting protein
		SO_2_ transporter	Cell division protein
			Clp protease subunit
**160**	3805	ARF	Arginine serine-rich splicing protein
**398**	4590	CytC oxidase	SOD
		CSD	Pollen protein
			Transcription factor lim1
**477**	8170	ND	Aldehyde dehydrogenase
			Clathrin heavy chain
**393**	8255	b-ZIP	Sphingolipid desaturase
		F-box	
**168**	10670	AGO	HSP interaction protein
**169**	15499	HAP2	Ring-finger protein
			26S proteasome subunit
			Galactosyltransferase
**2118**	16199	ND	Methyltransferase
			Ribosomal protein
			Vacuolar ATP synthase
**171/170**	20383	SCL	Protein kinase
			Methyltransferase protein
			Calcium ion binding
			Porin
**162**	21640	Dicer	Dipeptidyl peptidase
**167**	39273	ARF	ARF
			Cytochrome p450
			Vascular protein
**482**	61179	ND	Eukaryotic initiation factor
			Chalcone flavanone isomerase
**396**	73058	GRF	GRF
			Ubiquitin protease 2b
			Cyclin
**529**	75594	SBP	SBP
			Chloroplast chaperonin
			Serine protease
**156**	100279	SBP	SBP
			Cytochrome P450
**159/319**	748920	MYB	TCP
		TCP	sRNA methyltransferase
			Ribosome protein
			Reverse transcriptase
**165/166**	13078590	HD-ZIPIII	HD-ZIP III
			AP3-complex subunit
			RNA polymerase subunit
			Topoisomerase

1 The miRNA targets of *Arabidopsis thaliana* and *Oryza sativa* mainly consults reviewer papers [19], [41], [59].

2 The GI number of the putative targets were provided in the Additional file 4.

Abbreviations: 2-PGK, 2-phophoglycerate kinase; AP2,*?*APETALA2; APS, ATP-sulfurylase; ARF, auxin response factors; CytC, cytochrome; CSD, copper superoxide dismutase; AGO, ARGONAUTE; SCL, scarecrow-like; GRF, growth regulating factor; SBP, SQUAMOSA-promoter binding protein; ta-siRNA, *trans*-acting short interfering RNA; SOD, Superoxide Dismutase; PPR: pentatricopeptide repeat; HSP, heat shock protein.

**Figure 7:**
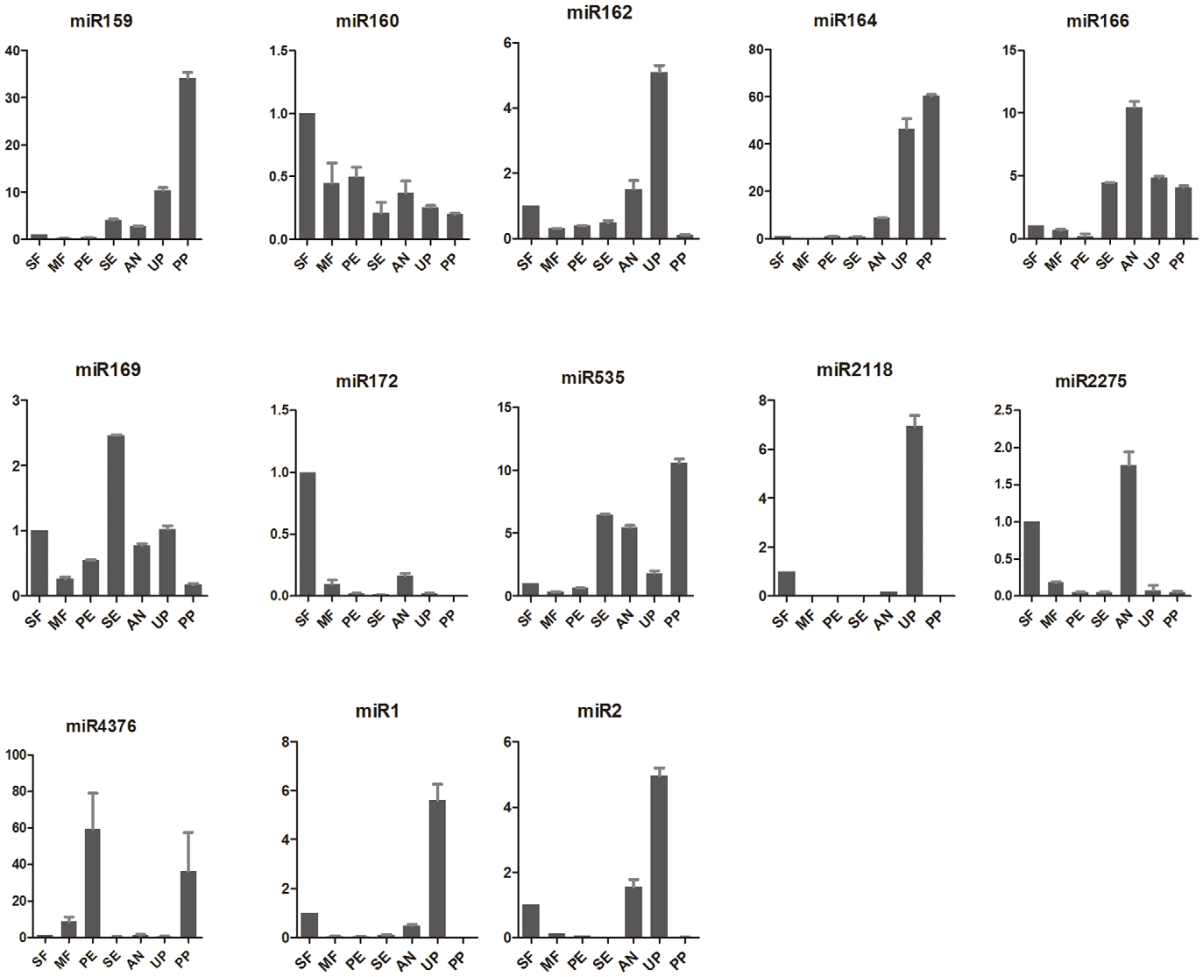
Relative expression level of some miRNAs in seven tissues. SF, small flower; MF, middle flower; PE, petal; SE, sepal; AN, anther; UP, unpollinated pistil; PP, pollinated pistil.

Since the miRNA library was constructed with *L. chinense* flowers at different developmental stages, a number of reproductive growth associated miRNAs were expected to be identified. MiR156, whose target is squamosa promoter binding protein-like (SBP or SPL) genes, has been reported to regulate the floral meristem identity [35]. Some other miRNAs, such as miR159, miR164 and miR172, have also been implicated to be involved in the regulation of flowering time and floral organ identity [36], [37], [38]. Recently, it was reported that miR4376 was also involved in the control of reproductive growth through regulating the expression of Ca^2+^-ATPase encoding gene *ACA10* in tomato [39]. Although miR164 and miR172 did not have the same target as in *Arabidopsis thaliana*, miR156 and miR159 had the same targets in both *L. chinense* and *Arabidopsis thaliana* (Table 3). To know if these miRNAs also regulate the reproductive growth in *L. chinense*, real-time PCR was conducted to check their expression in 7 different flower organs/tissues: small flower (SF) with 10.0–15.0 mm diameter, middle (MF) flower with 15.0–20.0 mm diameter, petal (PE), sepal (SE), anther (AN), unpollinated pistil (UP) and pollinated pistil (PP) from opened flowers (Fig. 7). The expression of some other miRNAs, such as miR162, miR165/166, miR169, miR535 and miR2118 were also checked here. Except for miR160 and miR169 that had constitutive expression, others expressed either tissue or stage specifically (Fig. 7), which implied these miRNAs and their targets might play important roles during the reproductive growth.

**Table 3:** Novel miRNAs in *L.chinense.*

miRNAsName	Mature Sequence	Length(nt)	Energy(kcal)	Readscount
lc-miR1	CAUGGCAGUAGAAGAGAUCACG	22	−30.23	22
lc-miR1*	UGAUCGCUUCUAUUGUUAUUUC	22	−30.23	15
lc-miR2	UCAACACUGAGGUCAUGGGUU	21	−24.94	105

MiR165/166 family was reported to play important roles in SAM (shoot apical meristem) development and their targets, HD-ZIP domain containing genes controlled leaf polarity and vascular differentiation [40]. Over-expression of miR166 could cause female sterility in Arabidopsis [41], which is consistent with our result that it had higher expression level in anther than in pistil (Fig. 7). In terms of anther or pistil specificity, miR172 and miR2275 had similar expression pattern with miR166. MiR172 was reported to be involved in the regulation of floral tissue identity through negatively regulating the expression of AP2 [34]. MiR2275 was recently reported as a new class of miRNAs that specifically expressed in rice stamen and could produce phased small RNA. This class of miRNAs is possibly conserved in gramineous plants like rice and maize [42], [43]. They could invoke a mass of siRNA (phased siRNAs) from dsRNA to regulate the inflorescence development of rice. Although the expression patterns of these three miRNAs were similar in both basal and evolved angiosperm plants, it is still an open and interesting question to know if they all function similarly.

Interestingly, miR2118, which was reported to be similar with miR2275 specifically expressed in rice stamen, was specifically expressed in the unpollinated pistils in *L. chinense*. It will be very interesting to know how its function evolved. Similarly expressed miRNAs included miR162 and miR169 (Fig. 7). To the contrary, the expression of miR159, miR535 and miR4376 were dramatically increased in pistil after pollination (Fig. 7). MiR159/319 family had been found to cleave the mRNAs of TCP and MYB transcription factors [41]. Over-expression of miR159 could cause male sterility in Arabidopsis [41], which is consistent with its low expression in anther (Fig. 7). It is very interesting to further study if the simultaneous highly expressed miR165/166 and miR159 in the flower of *L. chinense* had relation with its low setting percentage [44]. These miRNAs either increased or decreased their expression after pollination, which highly indicated that they might be involved in the regulation of pollination in *L. chinense*. But how these miRNAs or their target genes affect the pollination is still unknown. It will be worthy both theoretically and practically to explore their functions on controlling successful pollination in plants.

### Identification of Novel miRNAs in *L. chinense*

**Figure 8:**
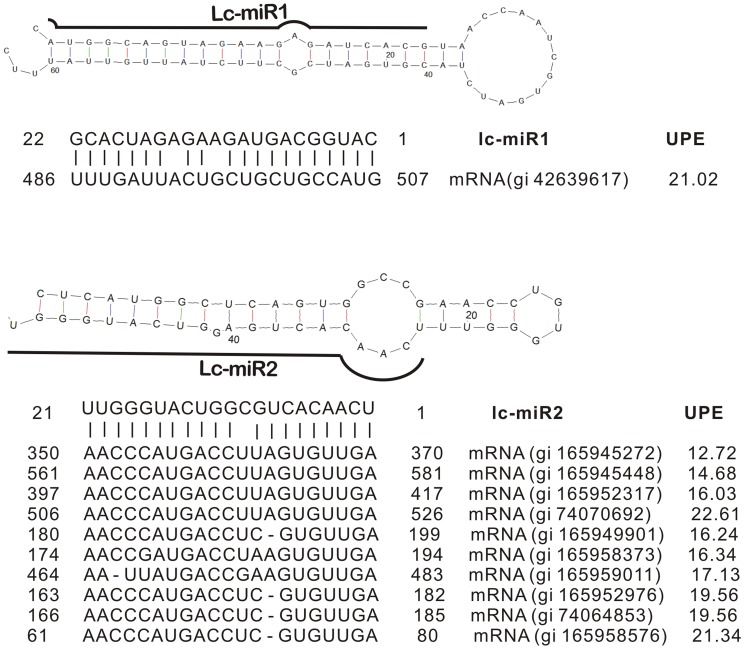
Putative stem-loop structures and target genes of novel miRNAs.

**Figure 9:**
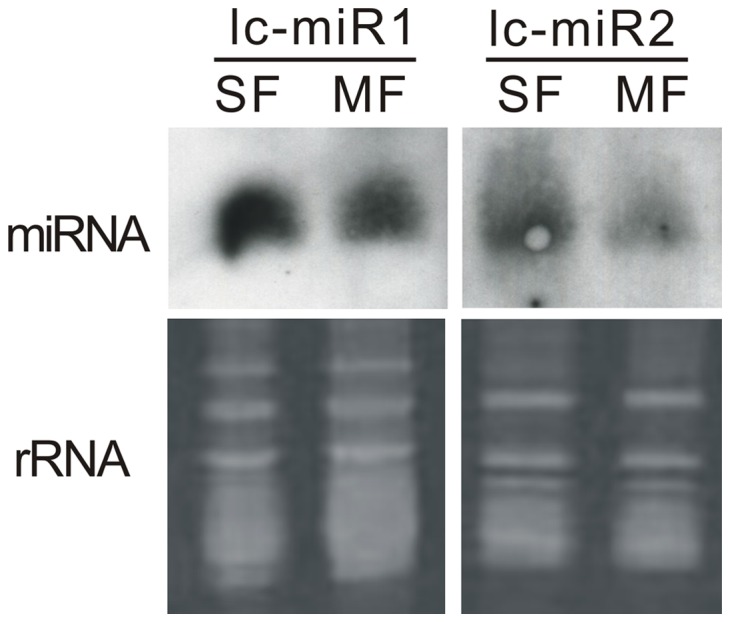
Northern blotting showing the expression of lc-miR1 and lc-miR2. rRNAs were used as loading control. SF, small flower; MF, middle flower.

*L. chinense* is an unsequenced species without any genomic information in the database. But there is an ESTs library available for its relative *L.tulipifera* [7]. In this study, we performed screening against *Liriodendron tulipifera* ESTs to predict precursor miRNAs in order to identify novel miRNAs. Using softwares miRDeep-P and miRNAFinder, we predicted several putative pre-miRNAs that could fold into classical stem-loop structures. Some of them had their mature transcripts detected in our sequencing data. To obtain a reliable result, only those with more than 20 reads were accepted as the real novel miRNAs. Based on these criteria, only 2 novel miRNAs were selected and designated as lc-miR1 and lc-miR2 (Table 3). The putative precursors from ESTs of *L. tulipifera* could fold into perfect stem-loop structures (Fig. 8). Lacking of genomic information might be the main reason that leads to the identification of only two novel miRNAs. Northern blotting was performed to validate the existence of these miRNAs. The expression of lc-miR1 and lc-miR2 could be detected in small flower (SF) and middle flower (MF) (Fig. 9&S1&S3). Both lc-miR1 and lc-miR2 had slight lower expression in MF than in SF (Fig. 9&S1&S3), which matched well with the real-time PCR results (Fig. 8). Furthermore, the expression of these two miRNAs and their putative target genes showed significant negative correlation (Fig. S1), which indirectly proved the existence of functional lc-miR1 and lc-miR2. Their mature sequences were aligned with all the miRNAs registered in the miRNA database miRBase (http://www.mirbase.org/) and PMRD (http://bioinformatics.cau.edu.cn/PMRD/), CSRDB (http://sundarlab.ucdavis.edu/smrnas/) and non-redundant sequences in Genebank. There was no homolog matching with them, which implied the two miRNAs found in our data are novel and might be Liriodendon-specific miRNAs. Both of them had high expression level in the un-pollinated mature pistil, whereas, both of their expression were sharply decreased after pollination (Fig. 7). This invoked us to analyze their target genes that might be important for the pollination. For miR1, one of the putative target genes encodes the large subunit of carbamoyl phosphate synthase (CPSase). The CPSase is a chloroplast protein responsible for the synthesis of carbamoylphosphate for both pyrimidine and arginine biosynthesis [45]. This is a highly controlled processes, and the transcription of CPSase encoding genes are sensitively invoked by ornithine and feedback down-regulated by pyrimidine or arginine [46]. But how the regulation happens is still unknown. The results about lc-miR1 suggested that miRNAs might at least used to be involved in the regulation in *L. chinense*. It has been reported that accumulation of pyrimidine and purine in the style happened after the pollination in Petunia [47]. The sharply decrease of lc-miR1 might up-regulate CPSase and enhance the biosynthesis of pyrimidine for pollen growth. For lc-miR2, we further analyzed the putative 10 target genes. The alignment among them showed that 5 transcripts had high sequence similarity in some regions (Figure S2). In coincidence, the lc-miR2 target sites on them also located in this putative conserved region. This implied that the lc-miR2 might regulate a multi-gene family in Liriodendrion. Blast analysis showed these transcripts had high similarity (61.15%) with Cysteine-rich receptor-like protein kinase (CRK) in *Medicago truncatula*. The CRK family in Arabidopsis was reported to be induced by pathogen infection, salicylic acid and reactive oxygen species [48], [49]. Till now, no literature reported that the CRK family might be involved in the pollination process. But the study on self-incompatibility in Brassicaceae indicated that the cysteine-rich-motif containing proteins like SRK and SCR were the important determinant factors in pollen recognition process [50]. Our findings in this paper indicate that the CRK proteins might be regulated by lc-miR2 during the pollination process in *L. chinense*. As mentioned above, *L. chinense* is endangering because of its low sexual reproductive efficiency. Up to now, it is still elusive about the molecular mechanism. The existence of these CRK like genes might provide a clue for us to further study the mechanism that leads to the low sexual reproductive efficiency in this species. The above analysis showed that lc-miR1 and lc-miR2 could be Liriodendron-specific miRNAs and play novel functions in this genus. It would be interesting that if this kind of regulation (through lc-miR1, lc-miR2 and their targets) also exists in other plants or not.

This is the first comprehensive analysis of miRNAs in *L. chinense*. A lot of conserved and novel miRNAs were identified. The data might be helpful not only in filling the gap of miRNA registered about basal angiosperm plants but also in understanding the evolution of miRNAs. Previous, Liang et al. have reported that many novel mRNAs were found in the *Liriodendron tulipifera* [51]. Our finding revealed that some novel miRNAs also existed in *L. chinense* and might be involved in the regulation of reproductive process. Exploring the regulatory mechanism of these miRNAs should be helpful to understand the sexual reproduction in these basal angiosperms. As an evolutionary important plant genus, Liriodendron might contain a certain amount of novel genes, including miRNA genes. With the availability of more genomic sequence information on Liriodendron genus, more miRNAs will be identified in this species. The cloning and identification of these genes and figuring out their regulation relationships would be very helpful for exploiting new genes and regulatory pathways and their evolution in plant.

## Materials and Methods

### Plant Material and Small RNAs Isolation

Young floral buds with the diameter of approximately 10.0–15.0 mm (small flower) and 15.0–20.0 mm (middle flower) in length and the petal (PE), sepal (SE), anther (AN), unpollinated pistil (UP) and pollinated pistil (PP) from open flowers of *L. chinense* were collected from wuhan botanical garden, wuhan city, China on Aprile 15, 2011, and then kept at −80°C. Total RNA was extracted from the different organs of flower with Trizol (invitrogen). After 15% polyacrylamide denaturing gel (8 M urea) electrophoresis, the small RNAs with size of 18–30 nt were excised from the gel and recovered by elution with 0.4 M NaCl overnight at 4°C. The elution solution was precipitated by adding three volume of absolute ethanol.

### Small RNA Library Construction and Sequencing

The small RNAs were dephosphorylated by alkaline phosphatase for avoiding self-ligation and then sequentially ligated 3′ and 5′ RNA/DNA chimeric oligos adapters. After reverse transcription and PCR, the amplified products were sequenced by Solexa GAII (Majorbio,shanghai,China).

### Discovery and Analysis of Conserved Micro RNAs

The raw sequencing data were filtered with software Fastx-Toolkit (http://hannonlab.cshl.edu/fastx_toolkit/) to delete the low quality reads, adapters and contamination. The clean reads were then annotated on Rfam 10.1 (http://rfam.sanger.ac.uk) to remove the rRNA, snRNA, snoRNA, tRNA. Then, the unique small RNAs filtered by Rfam were aligned with the data in miRBase 17 using Bowtie software (http://bowtie-bio.sourceforge.net/index.shtml) to search the conserved miRNAs in *L. chinense*. For the abundance analysis of miRNAs, the number of the read (read count) acquired by Solexa GAII was used.

### Novel miRNA Prediction

The de novo prediction of novel miRNA was performed by miRDeep-P (http://faculty. virginia.edu/lilab/miRDP/) and miRNAFinder(http://bioinfo3.noble.org/mirna/). The putative novel miRNAs were screened in our sequencing data. The putative miRNAs that had more than 20 reads matching with unique sequences were taken as candidates. Then the candidates were validated further by realtime PCR. Their putative stem-loop structures were showed with RNAdraw software.

### Prediction of miRNA Target

During the miRNA putative target analysis, psRNATarget (http://plantgrn.noble.org/psRNATarget/) [52] was used, the parameters were set as below: Maximum expectation: 3, Length for complementarity scoring: 20, Allowed maximum energy to unpair the target site (UPE): 25, Flanking length around target site for target accessibility analysis: 17 bp, Range of central mismatch leading to translational inhibition: 9–11 nt. Currently there are no genome sequence of *L. chinense* provided in NCBI. So we used the total ESTs of genus Liriodendron as the pool for the putative miRNA targets prediction. The conserved and novel miRNAs identified in this study were used as baits. The blast and GO annotation of miRNA targets were using Blast2go software [53].

### Northern Blotting for miRNAs

For Northern blot experiments, detailed procedures were referred to previous methods [54], [55]. Briefly, 50 ug of total RNA was size-fractionated through electrophoresis on 17% (w/v) denatured PAGE gel with 7 M urea. RNA was blotted on to Hybond-XL membranes (GE Healthcare) by semi-dry transfer unit (Bio-Rad). Hybridization probes for lc-miR1 (CGTGATCTCTTCTACTGCCATG), lc-miR1* (GAAATAACAATAGAAGCGATCA) and lc-miR2 (AACCCATGACCTCAGTGTTGA) were synthesized from HuiRui Biotech (Shanghai, China).

### Validation of miRNA Expression by Real-time PCR

The reverse transcription reaction and Real-time quantification was performed according to the protocol from chen [56]. The designed specific stem-loop primers and other primers for miRNAs arelisted in Table S1. No RT primer and no RNA controls were performed during reverse transcription. Real-time PCR was carried out on Lightcycler 480 system (Roche) with SYBR Green I methods. The melting curves were analyzed to check the specificity of PCR products. All the reactions were run for three replicates. The values of the threshold cycle (Ct, it was named as Cp in Roche Lightcycler 480 system) were calculated using Lightcycler 480 software. Because the different primer pairs showed different amplification efficiency, for comparision of gene expression in different tissues, the efficiency-calibrated method was used [57]. The 18S rRNA was used as internal reference and the expression of other tissues were relative to that of small flower (SF).

## Supporting Information

Figure S1 **The novel miRNAs and their targets expression in different tissues by qRT-PCR.**
(TIF)

Figure S2 **The aligment of 10 putative targets of lc-miR2.** The high similarity region between the target genes of lc-miR2 was showed as red box and the putative target region by lc-miR2 was showed as blue box.
(PDF)

Figure S3 **The expression of novel miRNAs in different tissues by Northern Blotting.** rRNAs were used as loading control. SF, small flower; MF, middle flower; PE, petal; SE, sepal; AN, anther; UP, unpollinated pistil.
(TIF)

Table S1 **The primers used in this study.**
(DOC)

Table S2 **The 496 conserved miRNAs represented 97 miRNA families in** ***L. chinense.***
(XLS)

Table S3 **The miRNAs targets predicted by psRNATarget and their GO analysis.**
(XLS)
