# Supplementary figures and images for: Identification of Conserved and Novel microRNAs from Liriodendron chinense Floral Tissues
Source: PLoS One. 2012 Sep 18;7(9):e44696. doi: 10.1371/journal.pone.0044696 (PMC3445533; doi:10.1371/journal.pone.0044696)

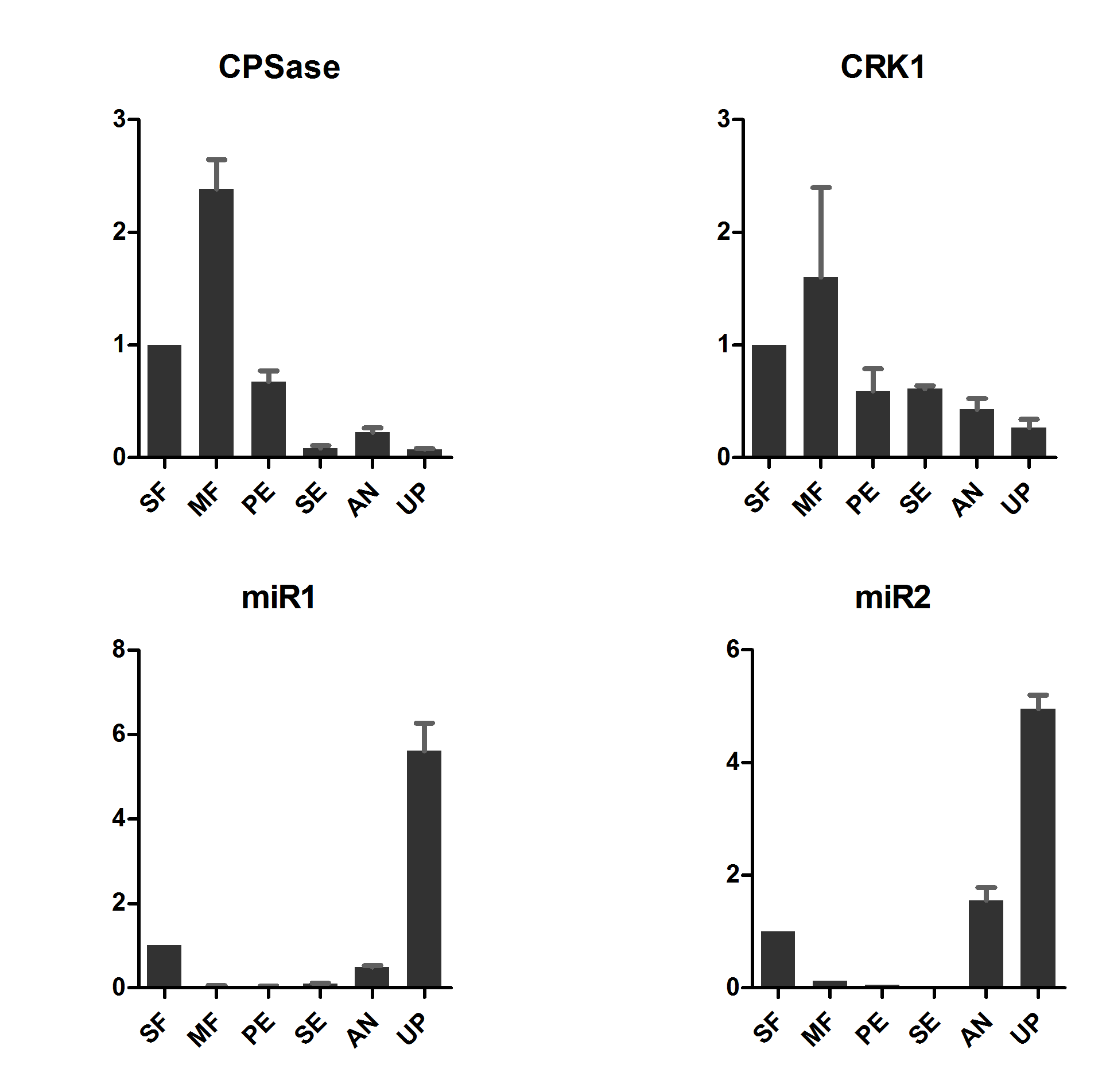

Supplement: Figure S1 — The novel miRNAs and their targets expression in different tissues by qRT-PCR. (TIF) [file pone.0044696.s001.tif]

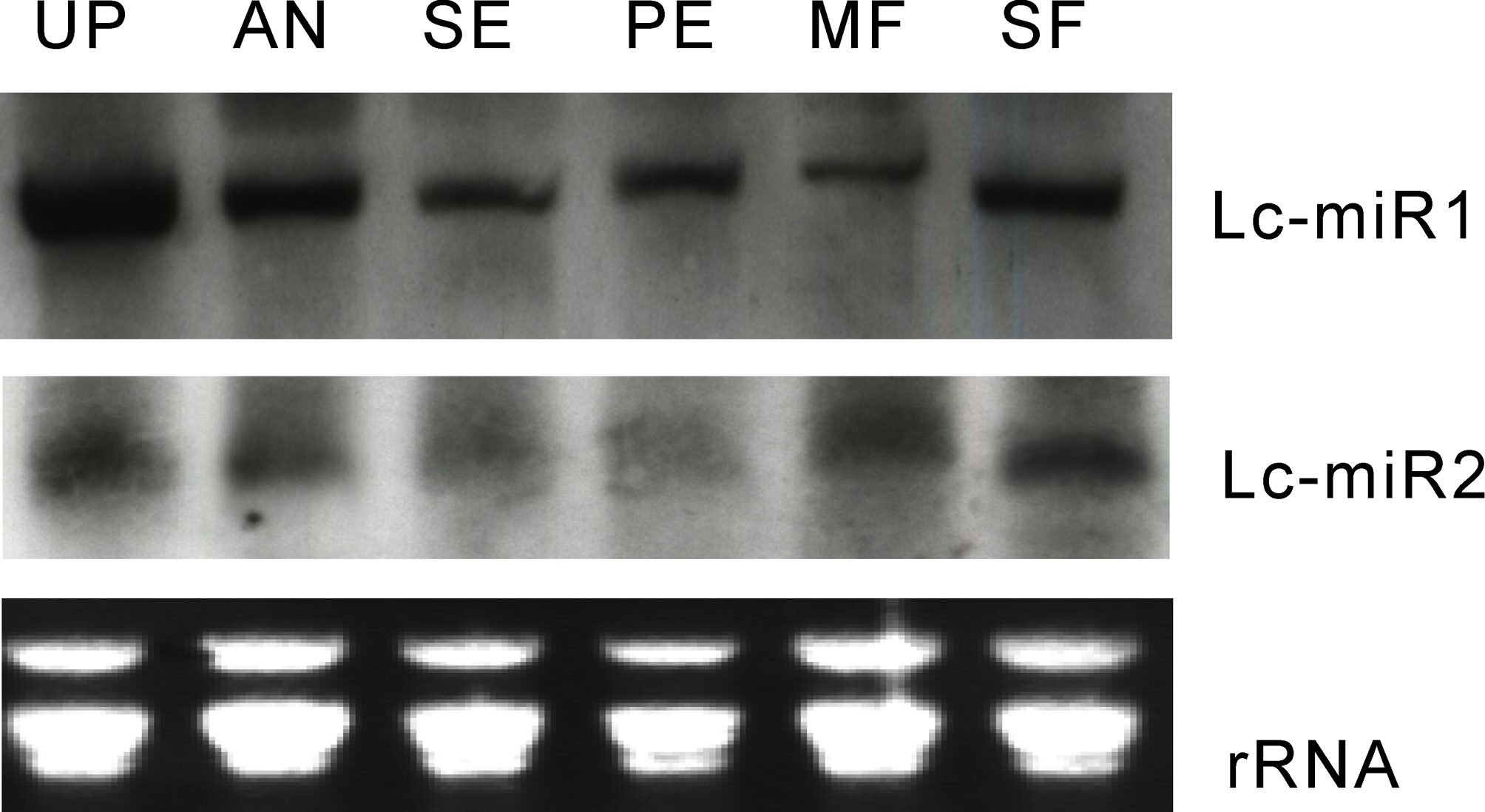

Supplement: Figure S3 — The expression of novel miRNAs in different tissues by Northern Blotting. rRNAs were used as loading control. SF, small flower; MF, middle flower; PE, petal; SE, sepal; AN, anther; UP, unpollinated pistil. (TIF) [file pone.0044696.s003.tif]
