# Supplementary material for: Identification of Conserved and Novel microRNAs from Liriodendron chinense Floral Tissues
Source: PLoS One. 2012 Sep 18;7(9):e44696. doi: 10.1371/journal.pone.0044696 (PMC3445533; doi:10.1371/journal.pone.0044696)
Supplement: Figure S2 — The aligment of 10 putative targets of lc-miR2. The high similarity region between the target genes of lc-miR2 was showed as red box and the putative target region by lc-miR2 was showed as blue box. (PDF) [file pone.0044696.s002.pdf]

```
                                                           Section 5
                                 (149) 149        160       170            185
gi_165945272_gb_FD487622.1_FD487622   (1) -------------------------------------
gi_165945348_gb_FD487675.1_FD487675 (148) ATTTGGCTGGGTGGGACTTCTCTCCTAGACTGAGTTC
gi_165949901_gb_FD490863.1_FD490863  (51) TCATATATTTTGTGTACACTGATATTTTGAGTGACAA
gi_165952976_gb_FD497722.1_FD497722  (43) TAATAAACTCT-TCCATCTTTCTTTCTT---------
  gi_74064853_gb_DT580075.1_DT580075  (46) TAATAAACTCT-TCCATCTTTCTTTCTT---------
gi_165958576_gb_FD501937.1_FD501937   (1) -------------------------------------
gi_165958373_gb_FD501509.1_FD501509  (50) TGCAACAAACCACCTAGGCAGGCTAGGTGAG------
gi_165952317_gb_FD493497.1_FD493497  (94) TCCAAAAGGAAAGCAATCAGAGGCTCAT--GGAAGGG
gi_165959011_gb_FD501138.1_FD501138 (121) GACCTAGGGTTATCCGAAGGACTTCTGTCAATATTGA
  gi_74070692_gb_DT585914.1_DT585914  (62) GCTTGAATTCTATGAGCAACAACCTTCTTAGCATCTT
                          Consensus (149) T  TAAA   T TC A      T T  T
                                                           Section 6
                                 (186) 186           200       210          222
gi_165945272_gb_FD487622.1_FD487622   (1) ---------------------------CCAAG--AGA
gi_165945348_gb_FD487675.1_FD487675 (185) AGTTCATGGTGCTGATGATCCCTTTCACCAAG--AGA
gi_165949901_gb_FD490863.1_FD490863  (88) GTATTACAGCCCCTTTTTTTTTACACACGCAC--TCA
gi_165952976_gb_FD497722.1_FD497722  (70) -TCTTCTTCTTCTTTTTTTTTTACACATGCAC--TCA
  gi_74064853_gb_DT580075.1_DT580075  (73) -TCTTCTTCTTCTTTTTTTTTTACACATGCAC--TCA
gi_165958576_gb_FD501937.1_FD501937   (1) --------------------------ACGCAC--TCA
gi_165958373_gb_FD501509.1_FD501509  (81) GTGTTCAGGAGAGTTGAGGCTAGCTAGTATAA--AGT
gi_165952317_gb_FD493497.1_FD493497 (129) GAGTTACTGTACATACTATTGAGCATATGAAC--ACA
gi_165959011_gb_FD501138.1_FD501138 (158) GTGTCCAGCAGCACGTGGTGGAGGGATCCAAGATTTA
  gi_74070692_gb_DT585914.1_DT585914  (99) -CCACCTTGCCAACTTGATGCTACTCTCCTCC--TTT
                          Consensus (186)  T TTC  G  C TTT  T    C CACG AC  TCA
                                                           Section 7
                                 (223) 223    230       240                259
gi_165945272_gb_FD487622.1_FD487622   (9) ATCCAAACAAGGGTTCAGATTGTAGATACGACATCCA
gi_165945348_gb_FD487675.1_FD487675 (220) ATCCAAACAAGGGTTCAGATTGTAGATACGACATCCA
gi_165949901_gb_FD490863.1_FD490863 (123) CACCCCACA----CTCACGCACTTG-CGCACCATAAT
gi_165952976_gb_FD497722.1_FD497722 (104) CACCCCACAAACACTCACGCAGT---CACACCACAGT
  gi_74064853_gb_DT580075.1_DT580075 (107) CACCCCACAAACACTCACGCAGT---CACACCACAGT
gi_165958576_gb_FD501937.1_FD501937  (10) CACCCCATA--------TGCACT---CACACCACAGT
gi_165958373_gb_FD501509.1_FD501509 (116) GGCCCTGATTTTATTTTTTTTTTAATTACA-CACCCA
gi_165952317_gb_FD493497.1_FD493497 (164) CAC-ATGCACGCACGCGCACACA------AACACGCA
gi_165959011_gb_FD501138.1_FD501138 (195) ATCCCCCATTTCTAATATTTTGTTGTGATGTCCCATT
  gi_74070692_gb_DT585914.1_DT585914 (133) ATCTGTGCTCGGGA-CTGGCAAT---GAGAGCGCAAA
                          Consensus (223) CACCCCACA     TCA GCAGT    ACA CACA T
                                                           Section 8
                                 (260) 260        270       280            296
gi_165945272_gb_FD487622.1_FD487622  (46) TGATGTGGACAGGTATTTGTAGTTTTTACAAAGATAA
gi_165945348_gb_FD487675.1_FD487675 (257) TGATGTGGACAGGTATTCGTAGTCTCTACAAAGATAA
gi_165949901_gb_FD490863.1_FD490863 (155) GGGATTTCACCACAATGGATACTTGAACCCATGACCT
gi_165952976_gb_FD497722.1_FD497722 (138) GGGATTTCACTACAATGGGTACTCAAACCCATGACCT
  gi_74064853_gb_DT580075.1_DT580075 (141) GGGATTTCACTACAATGGGTACTCAAACCCATGACCT
gi_165958576_gb_FD501937.1_FD501937  (36) GAGATTTCACCACAATGGGTACTCGAACCCATGACCT
gi_165958373_gb_FD501509.1_FD501509 (152) TGCACT---CCACAATGGGTACTTGAACCGATGACCT
gi_165952317_gb_FD493497.1_FD493497 (194) TGGAGTTCCCTCATATC--AAGTGAAAACACAGGCAT
gi_165959011_gb_FD501138.1_FD501138 (232) CCTAATATATCACTATACCCATGCCACCCTCGGACTT
  gi_74070692_gb_DT585914.1_DT585914 (166) GACTTGTTGGCGGAGTTAGATATCGAAA--ATGGCCG
                          Consensus (260) GGGATTTCACCACAATGGGTACTC AACC ATGACCT
```

```
                                                      Section 9
                                         (297) 297          310      320         333
gi_165945272_gb_FD487622.1_FD487622      (83)  ATTT---TTTTCAACTCAACAGAGTC----AGCTGGG
gi_165945348_gb_FD487675.1_FD487675      (294) ATCT---TCCTCAACTCAACAGAGTC----AGCTGGG
gi_165949901_gb_FD490863.1_FD490863      (192) CGTG---TTGA-AACTCTTGTGAGTCTACCATCCAGG
gi_165952976_gb_FD497722.1_FD497722      (175) CGTG---TTGA-AACTCTTGTGAGTCTACCACTGAGG
gi_74064853_gb_DT580075.1_DT580075       (178) CGTG---TTGA-AACTCTTGTGAGTCTACCACTGAGG
gi_165958576_gb_FD501937.1_FD501937      (73)  CGTG---TTGA-AACTCTTGTGAGTCTACTACCGAGG
gi_165958373_gb_FD501509.1_FD501509      (186) AAGT--GTTGA-ATCTCTTGTGAGTCTACCACTAAGC
gi_165952317_gb_FD493497.1_FD493497      (229) CTGA--GTTGGTGATGAGGTAAACACCACCATTAGAT
gi_165959011_gb_FD501138.1_FD501138      (269) GTGTGGAATTT-ACCTAAAAATACTTGA-GCTGAAGT
gi_74070692_gb_DT585914.1_DT585914       (201) CATTCAATTATGAGCTTAAAAGTGCCCAAGAAAGAAA
                                Consensus (297) C TT   TTGA AACTCTTGTGAGTCTACCA   AGG

                                                      Section 10
                                         (334) 334   340          350       360      370
gi_165945272_gb_FD487622.1_FD487622      (113) CATGTGATTGTTTTTTTCAGATGCTGGTTTTGGC--C
gi_165945348_gb_FD487675.1_FD487675      (324) CATGTGATTGTTTTTTTCAGATGCTGGTTTTGGC--C
gi_165949901_gb_FD490863.1_FD490863      (225) CATGAGTAAGGACC-TCTTGTTGAAACTTGTGAG--C
gi_165952976_gb_FD497722.1_FD497722      (208) CATGAGAGAGGACCCTCTTTTTAATACACTCTTC--C
gi_74064853_gb_DT580075.1_DT580075       (211) CATGAGAGAGGACCCTCTTTTTAATACACTCTTC--C
gi_165958576_gb_FD501937.1_FD501937      (106) CATGAGTATAAACC--CAAGATACGTTGAAGGCA--G
gi_165958373_gb_FD501509.1_FD501509      (220) CATGAGTAGGGACCCAAGCGGCCCTACTATTAAATGA
gi_165952317_gb_FD493497.1_FD493497      (264) CAATAAGTGGAAAGGATTTGCTCAAGTAGTTGTCTGC
gi_165959011_gb_FD501138.1_FD501138      (304) GGTGAGAAGGGATATAGATGCTTCACTTACTGAA-GA
gi_74070692_gb_DT585914.1_DT585914       (238) CACCTTTCCTACCCTTTGAGTTGAAGCATGTT----C
                                Consensus (334) CATGAGA  GGACC T  TG T CT CT TTG C  C
```

**The high similarity region between the target genes of lc-miR2 was showed as red box and the putative target region by lc-miR2 was showed as blue box.**
