# Supplementary material for: Identification of Conserved and Novel microRNAs from Liriodendron chinense Floral Tissues
Source: PLoS One. 2012 Sep 18;7(9):e44696. doi: 10.1371/journal.pone.0044696 (PMC3445533; doi:10.1371/journal.pone.0044696)
Supplement: Table S1 — The primers used in this study. (DOC) [file pone.0044696.s004.doc]

Table S

1 The primers sequences used in this study

| **miRNA** | **Function** | **Name and sequences** |
| --- | --- | --- |
| **Universal** | Reverse primer | >Reverse primer |
|  |  | GTGCAGGGTCCGAGGT |
| **miR2118** | RT primer | >2118p-RT |
|  |  | GTCGTATCCAGTGCAGGGTCCGAGGTATTCGCACTGGATACGACTCGGCA |
|  | Forward primer | >2118F |
|  |  | GGTTTCCGATGCCTCCCA |
| **miR2275** | RT primer | >2275a-RT |
|  |  | GTCGTATCCAGTGCAGGGTCCGAGGTATTCGCACTGGATACGACTGAGAT |
|  | Forward primer | >2275F |
|  |  | GCGCCTTTAGTTTCCTCCAAT |
| **miR1** | RT primer | >miR1-RT |
|  |  | GTCGTATCCAGTGCAGGGTCCGAGGTATTCGCACTGGATACGACCGTGAT |
|  | Forward primer | >miR1F |
|  |  | GCGCATGGCAGTAGAAGAG |
| **miR2** | RT primer | >miR2-RT |
|  |  | GTCGTATCCAGTGCAGGGTCCGAGGTATTCGCACTGGATACGAAACCCA |
|  | Forward primer | >miR2F |
|  |  | GCGCAACACGAGGCA |
| **miR159** | RT primer | >159-RT |
|  |  | GTCGTATCCAGTGCAGGGTCCGAGGTATTCGCACTGGATACGACCAGAGC |
|  | Forward primer | >159-F |
|  |  | GCGCCGGAGAAGGGA |
| **miR162** | RT primer | >162-RT |
|  |  | GTCGTATCCAGTGCAGGGTCCGAGGTATTCGCACTGGATACGACCTGGAT |
|  | Forward primer | >162-F |
|  |  | GGCCCGAAAACCCGC |
| **miR164** | RT primer | >164-RT |
|  |  | GTCGTATCCAGTGCAGGGTCCGAGGTATTCGCACTGGATACGACTGCACG |
|  | Forward primer | >164-F |
|  |  | GGGGAGAAGCAGGGCA |
| **miR166** | RT primer | >166-RT |
|  |  | GTCGTATCCAGTGCAGGGTCCGAGGTATTCGCACTGGATACGACGGGGAA |
|  | Forward primer | >166-F |
|  |  | CGCGGACCAGGCCA |
| **miR169** | RT primer | >169-RT |
|  |  | GTCGTATCCAGTGCAGGGTCCGAGGTATTCGCACTGGATACGACTCGGCA |
|  | Forward primer | >169-F |
|  |  | CGGCAGCCAAGGAGAC |
| **miR535** | RT primer | >535-RT |
|  |  | GTCGTATCCAGTGCAGGGTCCGAGGTATTCGCACTGGATACGACGCGTGC |
|  | Forward primer | >535-F |
|  |  | CGGCGACAACGAGAGAGA |
| **MiR160** | RT primer | >160-RT |
|  |  | GTCGTATCCAGTGCAGGGTCCGAGGTATTCGCACTGGATACGACTGGCAT |
|  | Forward primer | >160-F |
|  |  | GGUGCCUGGCUCCCUG |
| **MiR172** | RT primer | >172-RT |
|  |  | GTCGTATCCAGTGCAGGGTCCGAGGTATTCGCACTGGATACGACATGCAG |
|  | Forward primer | >172-RT |
|  |  | GGCGCCAGAAUCUUGAUGAUG |
| **MiR4376** | RT primer | >4376-RT |
|  |  | GTCGTATCCAGTGCAGGGTCCGAGGTATTCGCACTGGATACGACCAGTGT |
|  | Forward primer | >4376F |
|  |  | CGCACGCAGGAGAGATG |
| **18S** | Forward primer | CGTCCCTGCCCTTTGTACAC |
|  | Reverse primer | CGAACACTTCACCGGACCATT |
| **CPSase** | Forward primer | ACAGTTCCTCTTGGCTAA |
|  | Reverse primer | CTCTCAGTGGATGAAGTTG |
| **CRK1** | Forward primer | CTGAGTGCCTACATATAGTT |
|  | Reverse primer | CTTGGATGGTGCTCTTAT |
